# Roles of Lipid Peroxidation-Derived Electrophiles in Pathogenesis of Colonic Inflammation and Colon Cancer

**DOI:** 10.3389/fcell.2021.665591

**Published:** 2021-05-17

**Authors:** Lei Lei, Jianan Zhang, Eric A. Decker, Guodong Zhang

**Affiliations:** ^1^School of Medicine, Northwest University, Xi’an, China; ^2^Department of Food Science, University of Massachusetts, Amherst, MA, United States; ^3^Molecular and Cellular Biology Graduate Program, University of Massachusetts, Amherst, MA, United States

**Keywords:** inflammatory bowel disease, colonic inflammation, colorectal cancer, oxidative stress, lipid peroxidation

## Abstract

Redox stress is a common feature of gut disorders such as colonic inflammation (inflammatory bowel disease or IBD) and colorectal cancer (CRC). This leads to increased colonic formation of lipid-derived electrophiles (LDEs) such as 4-hydroxynonenal (4-HNE), malondialdehyde (MDA), trans, trans-2,4-decadienal (tt-DDE), and epoxyketooctadecenoic acid (EKODE). Recent research by us and others support that treatment with LDEs increases the severity of colitis and exacerbates the development of colon tumorigenesis *in vitro* and *in vivo*, supporting a critical role of these compounds in the pathogenesis of IBD and CRC. In this review, we will discuss the effects and mechanisms of LDEs on development of IBD and CRC and lifestyle factors, which could potentially affect tissue levels of LDEs to regulate IBD and CRC development.

## Introduction

Colonic inflammation (inflammatory bowel disease or IBD, including Crohn’s disease and ulcerative colitis) and colorectal cancer (CRC) are serious health problems in many countries. The incidence and prevalence of IBD have dramatically increased in the United States and other countries (Molodecky et al., 2012). The symptoms of IBD include abdominal pain, diarrhea, and rectal bleeding; as a result, IBD can severely impact the life quality of the patients. To date, there is no cure of IBD, and the current anti-IBD treatments can lead to serious side effects, such as increased infection risk, bone marrow dysfunction, organ dysfunction, and increased risk of malignancy, making it difficult to manage IBD. In addition, IBD patients have increased risks of developing CRC (Terzić et al., 2010). CRC is the third most common cancer and the second leading cause of cancer-related death worldwide (Bray et al., 2018). There are ~147,950 new cases of CRC in the United States in 2020. Although the majority of these cases occurred in individuals at an age of 50 years and older, ~12% new cases of CRC were diagnosed in individuals aged younger than 50 years (Siegel et al., 2020). It is of critical importance to better understand the pathological components involved in the development of IBD and CRC, in order to develop novel strategies for prevention and/or treatment.

A common feature of IBD and CRC is that the oxidative stress is increased in the colon tissues. Previous studies showed that a variety of reactive oxygen species (ROS), including superoxide (O_2_−), hydroxyl (OH), peroxyl (RO_2_), and alkoxyl (RO) radicals, are increased in the rodent models and human patients of IBD and CRC (Biasi et al., 2013). These ROS species can attack polyunsaturated fatty acids (PUFAs), notably linoleic acid (LA, the most abundant PUFA in humans diet and tissues), that are incorporated in the membrane phospholipids of colon tissues, leading to formation of endogenous lipid-derived electrophiles (LDEs), such as 4-hydroxynonenal (4-HNE), malondialdehyde (MDA), trans, trans-2,4-decadienal (tt-DDE), and epoxyketooctadecenoic acid (EKODE; Van Kuijk et al., 1990; Lin et al., 2007; Blair, 2008; Ayala et al., 2014). Substantial studies have shown that the levels of LDEs are increased in animal models and human patients with IBD or CRC (Skrzydlewska et al., 2005; Rezaie et al., 2007; Lee et al., 2010). In addition, previous studies have shown that the LDEs have potent effects on inflammation and tumorigenesis (Esterbauer et al., 1991). Therefore, some of the LDE compounds are implicated in the pathogenesis of IBD and CRC (Colgan and Taylor, 2010; Iborra et al., 2011; Bhattacharyya et al., 2014). However, most of the previous studies were performed using *in vitro* cell culture models (Esterbauer et al., 1991), which have several limitations: (1) the cell culture models have many limitations to study the complicated pathogenesis of IBD and CRC, (2) the LDEs are chemically reactive toward biomolecules and are metabolically unstable *in vivo*, the extent to, which these compounds can directly interact with intestinal epithelial cells (IECs) or immune cells *in vivo* remain unknown, and (3) some studies treated cultured cells with the LDE at high-μM concentrations, which may not be biologically or pathologically relevant.

**Figure 1:**
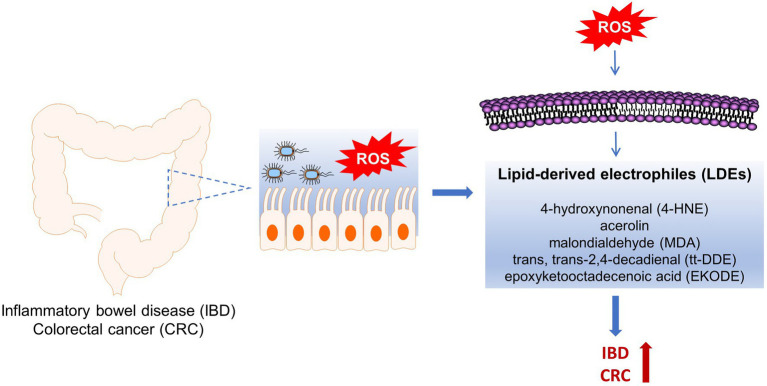
Roles of ROS-produced lipid-derived electrophiles (LDE) compounds in pathogenesis of inflammatory bowel disease (IBD) and colorectal cancer (CRC).

To address these concerns, recently we performed a series of animal studies to investigate the effects and mechanisms of LDEs, including 4-HNE, tt-DDE, and EKODE, on development of IBD and CRC in mouse models (Wang et al., 2019a, 2020; Lei et al., 2021). Our results showed that systematic, short-time, treatment with low doses of these compounds increased the severity of dextran sodium sulfate (DSS)-induced colitis and exacerbated the development of azoxymethane (AOM)/DSS-induced colon tumorigenesis in mice, supporting a critical role of these compounds in the development of IBD and CRC *in vivo* (Wang et al., 2019a, 2020; Lei et al., 2021). In this review, we will discuss the roles of the LDEs in the pathogenesis of IBD and CRC, and the implications of LDEs in designing strategies to reduce the risks of IBD and CRC (Figure 1).

## Levels of LDEs in Animal Models and Human Patients of IBD and CRC

Previous studies have shown that the concentrations of LDEs are increased in animal models of IBD (Table 1). In both DSS-and 2,4,6-trinitrobenzenesulfonic acid (TNBS)-induced colitic models, the concentration of 4-HNE is significantly increased in the colon tissues of colitic mice (Lee et al., 2010). Indeed, the colonic concentrations of free-form 4-HNE in normal C3H/HeN mice (not stimulated with DSS) vs. DSS-exposed C3H/HeN mice were 0.86 ± 0.85 ng/ml vs. 11.92 ± 7.01 ng/ml, demonstrating a dramatic increase of colonic 4-HNE in colitis (Lee et al., 2010). The concentration of MDA, another LDE compound, was also increased in the colon tissues of TNBS-induced colitic rats (Liu and Wang, 2011). These effects seemed to be mouse strain-dependent: DSS exposure significantly increased colonic concentration of 4-HNE in both C3H/HeN and C3H/HeJ mice, but the effect was much more dramatic in C3H/HeN mice compared with C3H/HeJ mice (Lee et al., 2010). Overall, these results support that the colonic concentrations of LDEs are increased in animal models of IBD.

**Table 1:** Concentrations of LDE compounds in animal models of IBD and CRC.

Model	Species	Tissue	Results	References
DSS/TNBS-induced colitis	C3H/HeN or C3H/HeJ mouse	Colon	↑ MDA, ↑ 4-HNE in colon	Lee et al., 2010
TNBS-induced colitis	Sprague-Dawley rat	Colon	↑ MDA in colon	Liu and Wang, 2011
Colitis-CRC model	*Il-10*^−/−^ mouse colonized with *Enterococcus faecalis* OG1RFSS	Colon	↑ 4-HNE-protein adducts in colon	Wang et al., 2012
AOM/DSS-induced CRC model	C57BL/6 mouse	Colon	↑ EKODE in colon	Lei et al., 2021
AOM/DSS-induced CRC model	C57BL/6 mouse	Colon	↑ MDA in colon	Amerizadeh et al., 2018
AOM/DSS-induced CRC model	BALB/c mouse	Colon	↑ MDA, ↑ 4-HNE protein in colon	Cid-Gallegos et al., 2020

AOM, azoxymethane; DSS, dextran sodium sulfate; TNBS, 2,4,6-trinitrobenzenesulfonic acid.

Previous studies also showed that the concentrations of LDEs are increased in animal models of CRC (Table 1). Our recent research showed that EKODE, an aldehyde compound derived from oxidative degradation of ω-6 PUFAs (Lin et al., 2007), was increased in the colon tissues of AOM/DSS-induced CRC mice (Lei et al., 2021). In our research, we used a liquid chromatography-tandem mass spectrometry (LC-MS/MS)-based metabolomics, which can measure >100 fatty acid metabolites derived from both enzymatic metabolism and non-enzymatic oxidation of PUFAs (Wang et al., 2019b), to systematically profile how fatty acid metabolites are deregulated in the colon of AOM/DSS-induced CRC mice. We found that EKODE was significantly increased in the colon of the AOM/DSS-induced C57BL/6 mice compared with that of the healthy control mice. In addition, EKODE was also among the most dramatically increased fatty acid metabolites in the colon of the mice (Lei et al., 2021). The concentration of EKODE was not significantly increased in the plasma of AOM/DSS-induced CRC mice compared with the healthy control mice (Wang et al., 2019b), and this could be due to the low chemical and/or metabolic stability of EKODE in circulation. Besides the chemically induced CRC models, the levels of LDEs are also increased in the CRC model of the *Il-10*^−/−^ mice. Compared with *Il-10*^−/−^ mice colonized with a superoxide-deficient strain WY84SS or administered sham, the *Il-10*^−/−^ mice colonized with a superoxide-producing *Enterococcus faecalis* strain OG1RFSS developed more severe colon tumorigenesis. Immunohistochemical analyses showed that the levels of 4-HNE-protein adducts are increased in the colonic macrophages and myofibroblasts of *Il-10*^−/−^ mice colonized with OG1RFSS (Wang et al., 2012). These results are consistent with other studies, which showed that LDE compounds, such as 4-HNE and MDA, are increased in animal models of CRC (Amerizadeh et al., 2018; Cid-Gallegos et al., 2020).

Human studies also showed that the concentrations of LDEs are increased in IBD and CRC patients (Table 2). Previous studies showed that the circulating concentration of MDA was increased in Crohn’s disease patients compared with control subjects (Alzoghaibi et al., 2007; Boehm et al., 2012; Achitei et al., 2013). The concentrations of 4-HNE and MDA are increased in human primary CRC tissues (Skrzydlewska et al., 2005). In addition, previous studies showed that CRC patients, as well as patients with unresectable colorectal liver metastasis, have higher concentrations of MDA in the urine and/or plasma (Saygili et al., 2003; Leung et al., 2008; Chandramathi et al., 2009). After surgical treatments, the serum concentration of MDA was reduced in CRC patients compared to presurgical status (Surinėnaitė et al., 2009). Clinical studies also showed a strong association between LDEs and transforming growth factor β1 (TGF-β1) levels, related to the tumor malignancy (Tüzün et al., 2012). 4-HNE may make an important contribution toward upregulating TGF-β1 expression (Leonarduzzi et al., 1997). Overall, these results support the clinical importance of LDEs in IBD and CRC.

**Table 2:** Concentrations of LDE compounds in human patients of IBD and CRC.

Disease	Human subjects	Tissue	Results	References
IBD	IBD patients (*n* = 42) and normal adults (*n* = 32)	Plasma	↑ MDA in plasma	Alzoghaibi et al., 2007
IBD	IBD patients (*n* = 41) and normal adults (*n* = 18)	Serum	↑ MDA in serum	Achitei et al., 2013
IBD (CD)	CD patients (*n* = 52) and healthy adults (*n* = 99)	Plasma	↑ MDA in plasma	Boehm et al., 2012
CRC	CRC patients (*n* = 81) and most distant location as control (*n* = 81)	Colon mucosae	↑ MDA, ↑ 4-HNE in colon mucosae	Skrzydlewska et al., 2005
CRC	CRC patients (*n* = 49) and healthy individuals control (*n* = 95)	Urine	↑ MDA in urine	Chandramathi et al., 2009
CRC	CRC patients (*n* = 20) and healthy individuals control (*n* = 20)	Plasma	↑ MDA in plasma	Saygili et al., 2003
CRC	Primary operable patients (*n* = 53) and advanced inoperable patients (*n* = 53)	Plasma	↑ MDA in plasma	Leung et al., 2008
CRC	CRC patients (*n* = 65; comparing the change between presurgical and postsurgical periods)	Serum	↓ MDA in serum compared to presurgical	Surinėnaitė et al., 2009

CD, Crohn’s disease; TBARS, thiobarbituric acid-reactive substances; UC, ulcerative colitis.

The increased colonic concentration of LDEs in IBD and CRC could be due to the more severe redox stress in these diseases. Substantial studies have shown that a series of oxidative markers, such as ROS species, nitric oxide, 8-oxo-2'-deoxyguanosine (8-oxodG), and antioxidant or pro-oxidative proteins (e.g., catalase and myeloperoxidase), are altered in IBD and CRC, demonstrating a more severe oxidative microenvironment in IBD and CRC (Perse, 2013; Sies et al., 2017). In agreement with these studies, we showed that compared with control healthy mice, the AOM/DSS-induced CRC mice had a lower colonic expression of a series of anti-oxidative genes, such as *Sod1* (encoding superoxide dismutase 1), *Cat* (encoding catalase), *Gsr* (encoding glutathione-disulfide reductase), *Gsta1* (encoding glutathione S-transferase A1), *Gstm1* (encoding glutathione S-transferase M1), and *Hmox1* (encoding heme oxygenase-1), and had higher colonic expression of a pro-oxidative gene *Mpo* (encoding myeloperoxidase), demonstrating more severe redox stress in the colon tissues of AOM/DSS-induced CRC mice. Consistent with these findings in animal models, we found that, in the Cancer Genome Atlas (TCGA) database, the expressions of the anti-oxidant genes (*CAT*, *GSR*, *GSTA1*, *GSTM1*, and *HMOX1*) are reduced, while the expression of pro-oxidant gene *MPO* is increased, in the tumor samples of human CRC patients (Lei et al., 2021).

The more severe redox stress in the colon tissues of IBD and CRC could contribute to the high concentrations of LDEs through several mechanisms. First, the colon tissues of IBD and CRC usually have higher levels of ROS species, which can directly attack membrane phospholipids and lead to increased production of LDEs such as 4-HNE and EKODE (Lin et al., 2007). Second, the colon tumors or inflamed colons usually have lower expression of glutathione S-transferases (GST), as well as glutathione, and this could lead to decreased metabolism of LDEs and thus contribute to their high abundance in colon tissues. Indeed, previous studies showed that the colonic concentration of glutathione, as well as the colonic activity of GST enzymes, was reduced in DSS-induced colitic model (Oz et al., 2005; Arafa et al., 2009). The GST activity in the distal colon was significantly lower in the carcinoma patients compared with the adenoma patients and healthy controls (Grubben et al., 2006). The GST enzymes are the major enzymes involved in metabolism of lipid peroxidation-derived α,β-unsaturated carbonyl compounds, such as 4-HNE and acrolein, catalyzing the conjugation reaction of these compounds with glutathione to form the corresponding glutathione conjugates (Allocati et al., 2018). Therefore, reduced expression of GST and lower levels of intracellular glutathione in IBD and CRC could lead to attenuated metabolic degradation of the LDEs. Overall, due to the oxidative stress in the colon tissues of IBD and CRC, there could be enhanced production and/or reduced degradation of LDEs in the colon, leading to higher colonic levels of LDEs.

## Effects of LDEs on Development of IBD and CRC

### *In vitro* Studies of LDEs on Inflammation and Tumorigenesis

Previous studies by us and others showed that treatment with low-concentration LDE increases inflammatory responses. In our recent study, we treated human CRC (HCT-116) and mouse macrophage (RAW 264.7) cells with EKODE, at a concentration of 300 nM (a dose determined from LC-MS/MS analysis of the concentration of endogenous EKODE in the colon tissues of AOM/DSS-induced CRC mice, see our publication Lei et al., 2021). We found that EKODE treatment induces the expression of pro-inflammatory genes and activates JNK and NF-κB pathways in both CRC and macrophage cells, illustrating a potent pro-inflammatory effect of EKODE *in vitro* (Lei et al., 2021). Besides EKODE, previous studies by us and others also showed that other LDE compound, such as tt-DDE, induces inflammatory responses *in vitro* (Chang et al., 2005; Wang et al., 2020).

Previous studies also support that LDEs can cause detrimental effects on tumorigenesis *in vitro*. Many LDE compounds, such as 4-HNE, MDA, and acrolein, are chemically reactive and can form covalently-linked conjugates with biomolecules such as DNA, leading to mutagenesis and tumorigenesis. Acrolein, a major component in cigarette smoke, has been shown to be able to directly react guanine residues in DNA to produce DNA adducts (Comes and Eggleton, 2002). It could be a major etiological agent for cigarette smoke-related lung cancer and contributes to lung carcinogenesis through the induction of DNA damage and the inhibition of DNA repair (Feng et al., 2006a). MDA has also been shown to react with nucleosides, such as deoxyguanosine and deoxyadenosine, to form adducts such as pyrimido[1,2-a]purin-10(3H)-one (M1G; Niedernhofer et al., 2003). M1G has been demonstrated to be highly mutagenic in human cells and has been detected in tissues under oxidative stress (Marnett, 1999a,b). MDA treatment inhibits nucleotide excision repair for both UV light-and BPDE-induced DNA damage in CRC cells (Feng et al., 2006b). These results suggest that MDA could play a critical role in oxidative stress-induced mutagenesis and carcinogenesis through two detrimental mechanisms: the induction of DNA damage and the inhibition of DNA repair. Besides MDA, other LDE compound, such as 4-HNE, has been shown to a potential mutagen and could contribute to oxidative stress-induced carcinogenesis (Hu et al., 2002; Nair et al., 2006; Wang et al., 2012, 2015). The effects of LDEs on inflammation and tumorigenesis have been summarized and discussed in several reviews (Poli et al., 2008; Ayala et al., 2014; Zhong and Yin, 2015) and will not be discussed in detail here.

Previous studies showed that LDEs can cause different, or even opposite, effects at different concentrations *in vitro*. For example, EKODE at a concentration of 10 μM can activate nuclear factor erythroid 2-related factor 2 (Nrf2) signaling (Wang et al., 2009), which is an important pathway involved in cellular defense against oxidative stress (Guina et al., 2015). While at lower concentrations, EKODE did not have such an effect and instead induced inflammatory responses (Wang et al., 2009; Lei et al., 2021). This could be, at least in part, due to the mode of actions of these compounds. LDEs are chemically reactive and can covalently modify cellular proteins, it is feasible that at different concentrations, the LDE compound can interact with different cellular proteins: at low concentrations, the LDE compound could selectively interact with the cellular proteins, which have the most reactive amino acid residues; while at high concentrations, the LDE compound could interact with more proteins in a less selective manner, resulting in varied or even opposite biological responses. This notion is supported by previous studies of click chemistry-based imaging of LDE compounds such as 4-HNE and tt-DDE (Vila et al., 2008; Wang et al., 2020). To better understand the biological effect of LDE compound, it is important to perform cell culture studies using a dose that is biologically or pathologically relevant. As we discussed in “Levels of LDEs in Animal Models and Human Patients of IBD and CRC” section above, substantial studies have reported the concentrations of endogenous LDEs in animal models and human patients of IBD and CRC: for example, previous studies showed that the colonic concentrations of 4-HNE are ~11.9 ng/ml (~76 nM) in DSS-exposed C3H/HeN mice and ~15.9 ng/ml (~102 nM) in TNBS-exposed C3H/HeN mice (Lee et al., 2010). These reported concentrations can help us to perform *in vitro* studies to study the actions of these compounds under biologically relevant conditions.

### *In vivo* Studies of LDEs on IBD and CRC

Our recent research showed that systematic, short-time, treatment with low doses of the LDEs, such as 4-HNE, tt-DDE, or EKODE, increased the severity of DSS-induced colitis and exacerbated the development of AOM/DSS-induced CRC in mouse models, supporting critical roles of these compounds in the development of IBD and CRC (Wang et al., 2019a, 2020; Lei et al., 2021). In our experiment, we treated C57BL/6 mice with 2% DSS in drinking water, with or without administration of 4-HNE, tt-DDE, or EKODE (*via* intraperitoneal injection, dose = 1–5 mg/kg/day), for 6–7 days, then sacrificed the mice for analysis. We found that LDE treatment increased the severity of DSS-induced colitis in mice, with increased infiltration of immune cells, expression of pro-inflammatory genes, and enhanced crypt damage, in the colon tissues. Furthermore, we showed that LDE treatment exacerbated intestinal barrier dysfunction, leading to enhanced translocation of bacteria or toxic bacterial products from the gut into the systemic circulation and distant organs. Overall, these results support that LDEs have a pro-colitic activity *in vivo* (Wang et al., 2019a, 2020; Lei et al., 2021). In addition, we also found that LDE treatment exacerbated the development of AOM/DSS-induced colon tumorigenesis in mice. In this experiment, we stimulated AOM and DSS to initiate colon tumorigenesis, then treated the mice with an intraperitoneal injection of EKODE (dose = 1 mg/kg/day). EKODE treatment increased tumor number and tumor size, and increased expression of pro-inflammatory and pro-tumorigenic markers in the colon, demonstrating its CRC-enhancing effects *in vivo* (Lei et al., 2021).

TLR4 plays a critical role in gut bacteria-host interactions by recognizing LPS, which is expressed by Gram-negative bacteria and certain Gram-positive bacteria (Abreu, 2010). Activation of TLR4 contributes to the development and maintenance of inflammatory responses (O’Shea and Murray, 2008), and the expression of TLR4 is significantly upregulated in the colon of DSS-induced colitic mice (Hou et al., 2013). TLR4 is generally expressed at the basolateral surface of intestinal epithelial cells; as a result, TLR4 signaling will only be activated when the gut bacteria penetrate the intestinal epithelium layer (Kubinak and Round, 2012). We found that in the DSS-induced colitic model, treatment with 4-HNE suppressed expression of tight-junction proteins in colon tissues, leading to increased translocation of bacteria or bacterial product (e.g., LPS) from the gut into the systemic circulation, resulting in increased activation of TLR4 signaling *in vivo* (Wang et al., 2019a). Furthermore, we showed that 4-HNE failed to promote DSS-induced colitis in *Tlr4*^−/−^ mice, supporting that TLR4 signaling is required for the pro-colitic activity of 4-HNE (Wang et al., 2019a). Our finding is consistent with previous studies, which showed that 4-HNE induces the production of pro-inflammatory cytokines (IL-8, IL-1β, and TNFα) and upregulates matrix metalloproteinase-9 by TLR4/NF-κB-dependent mechanisms *in vitro* (Gargiulo et al., 2015). Besides 4-HNE, we also found that EKODE treatment impaired intestinal barrier function and enhanced bacterial translocation *in vivo*, which could lead to activation of TLR4 signaling and contribute to its pro-colitic activity (Lei et al., 2021).

The potential roles of TLR4 in mediating the pro-colitic actions of LDEs suggest that gut microbiota could be involved in the actions of LDEs. The LDE compounds, which are increased in the colon tissues under IBD or CRC status, could directly interact with bacterial cells that reside in the colon, leading to alteration of gut microbiota and contributing to increased development of IBD or CRC (Sekirov et al., 2010). A healthy gut is a mostly oxygen-free environment and is mainly inhabited by obligate anaerobes (Sekirov et al., 2010). Previous studies have supported the notion that many beneficial gut bacteria are sensitive to oxygen or redox stress, while the pathologic bacteria are more resistant to redox stress (Sekirov et al., 2010). Therefore, increased formation of LDEs, which are chemically and redox-active, could perturb gut microbiota through suppressing the growth of beneficial anaerobic bacteria and enhancing the growth of redox-resistant pathological bacteria, resulting in microbiota dysbiosis. To date, few studies have characterized the roles of LDEs on gut microbiota, and the functional roles of the altered microbiota in promoting IBD or CRC. Further studies are needed to better understand how IBD or CRC-associated redox microenvironment interacts with the gut microbiota to affect the development of gut diseases.

Overall, our results support a model that during the development of colitis and CRC, there is enhanced production of ROS species in the colon, leading to increased production of LDEs such as 4-HNE and EKODE in the colon tissues. These compounds could interact with the cells that reside in the gut, such as IECs, inflammatory cells, or even gut bacteria cells, leading to increased inflammatory responses and tumorigenesis, and resulting in increased development of IBD and CRC. In support of this notion, the results by us and others showed that treatment with LDEs, at pathologically relevant concentrations, induced inflammatory responses in IECs and macrophages (Wang et al., 2019a, 2020; Lei et al., 2021). Therefore, these compounds could be important pathological components in the development of IBD and CRC. Future studies are needed to determine whether we could develop strategies to selectively target LDEs to reduce the risks of IBD and CRC.

We want to point out that our animal studies have limitations. The purpose of our research is to study the extent to, which LDE compounds modulate the development of colitis and CRC in mouse models. Since, the LDEs are produced by non-enzymatic oxidation of tissue PUFAs, it is difficult for us to use genetically engineered mouse models to alter colonic concentrations of LDEs and study their biological actions. In our experiments, we treated mice with these compounds, such as 4-HNE and EKODE (dose = 1–5 mg/kg/day), *via* intraperitoneal injection (Wang et al., 2019a, 2020; Lei et al., 2021). We used this dose range, since a previous study has shown to intraperitoneal injection of 5 mg/kg/day 4-HNE caused no toxic effects in mice (Nishikawa et al., 2000). However, intraperitoneal injection of LDEs leads to systematic delivery of LDEs and could also increase the concentrations of LDEs in other tissues. In addition, it remains to determine whether the colonic concentrations of LDEs in the LDEs-treated mice are relevant with those in IBD and CRC patients.

## Factors That Affect the Formation of LDEs in Tissues

Since, LDEs are produced from tissue PUFAs (notably LA) by the actions of ROS, factors that can affect the formation of LDEs in tissues, such as dietary intake of LA, heme irons, and antioxidants, could modulate the risks of IBD and CRC. The details are discussed below.

### Dietary Intake of LA

LA is abundant in vegetable oils, such as corn, soybean, and canola oils, as well as fried food, salad dressing, and mayonnaise (Blasbalg et al., 2011). Since the last century, there has been a dramatic increase of dietary consumption of LA in the United States and other countries: the consumption of soybean oil, which is a major vegetable oil on the market, has risen more than 47% since 1980 and more than 1,000-fold since 1909 (Blasbalg et al., 2011). It is feasible that a high intake of dietary LA would increase the abundance of LA in membrane phospholipids and leads to increased formation of LDEs under redox stress, which could result in increased development of IBD and CRC. In consistent with this notion, animal experiments showed that a high intake of LA increased both AOM-and *Apc* mutation-induced CRC, suggesting its potential adverse effect on CRC (Reddy et al., 1985; Wu et al., 2004; Fujise et al., 2007; Enos et al., 2016; Liu et al., 2020). Human studies also support that a high intake of LA increases the risks of CRC (Pot et al., 2008; Daniel et al., 2009) and colitis (Shoda et al., 1996; Tjonneland et al., 2009; Strassburg et al., 2014; Rashvand et al., 2015). Notably, the European Prospective Investigation into Cancer and Nutrition (EPIC) study showed that high intake of LA more than doubled the risks of IBD and could be responsible for ~30% of ulcerative colitis cases (Tjonneland et al., 2009), though we need to point out there are also inconsistent studies, which showed that a high dietary intake of LA did not increase risks of CRC in human populations (Zock and Katan, 1998; Bartsch et al., 1999; Azrad et al., 2013). Further studies are needed to better characterize the molecular mechanisms for the potential CRC-enhancing effects of dietary LA, in order to clarify its health effects and make dietary recommendations or guidelines for the optimal intake of LA.

### Dietary Intake of Heme Iron

Overall meat consumption has continued to rise in the United States and the rest of the developed world. Red meat represents the largest proportion of meat consumed in the United States (58%; Daniel et al., 2011). Heme has been proposed as the key molecule contributing to tumorigenesis upon red and processed meat intake (Fiorito et al., 2020). Heme iron plays important role in lipid peroxidation. Previous studies support that a high dietary intake of heme iron increases tissue levels of LDEs, leading to increased risks of CRC. Heme iron can increase lipid peroxidation in food products: Gasc et al. (2007) showed that heme iron can interact with dietary LA, leading to increased levels of 4-HNE in food products. In addition, Pierre et al. showed that administration of a diet rich in heme iron increased the urinal concentration of 1,4-dihydroxynonane mercapturic acid (DHN-MA), which is a major urinary metabolite of 4-HNE, in both animal models and human subjects, suggesting that dietary intake of heme iron increases lipid peroxidation *in vivo* (Pierre et al., 2006). Animal and human studies also support that heme iron increases risks of CRC. Feeding of a diet rich in heme iron increased the number of preneoplastic lesions in an AOM-induced CRC model in rats and increased tumor load in a spontaneous CRC model in *Apc^Min^* mice (Bastide et al., 2015). A meta-analysis of cohort studies showed that high heme iron intake was associated with increased risks of CRC, supporting that heme iron increases risks of CRC in humans (Bastide et al., 2011).

### Dietary Intake of Antioxidants

Radical scavenging antioxidants, which counteract the detrimental actions of ROS species and are used to inhibit lipid peroxidation in food products, are widely regarded to be beneficial. Previous studies support that some naturally occurring antioxidants, such as lycopene, flavonoids, phenolic, and polyphenolic compounds, have anti-tumor effects (Khurana et al., 2018) and could attenuate the risks of IBD and CRC (Murtaugh et al., 2004; Moura et al., 2015). Administration of antioxidant has been shown to reduce tissue levels of LDE compounds, such as MDA or its DHA adduct M1G, supporting a link of antioxidant intake with LDE compounds (Sharma et al., 2001; Vezza et al., 2016). However, there are recent studies that suggest that antioxidants can increase the risks of cancers in animal models and human subjects. Gallic acid, which is a phenolic acid widely found in plants, has been shown to increase the risks of CRC in *Apc^Min/+^p53^R172H^* mice (*Apc^Min/+^* mice with *p53* mutation), while it had no effects on *Apc^Min/+^* mice that express wild-type *p53* (Kadosh et al., 2020). In other types of cancers, Sayin et al. showed that dietary administration with the antioxidants, N-acetylcysteine (NAC) and vitamin E, markedly increased tumor progression and reduced survival in mouse models of B-RAF‐ and K-RAS-induced lung cancer (Sayin et al., 2014). Tumor metastasis, which is a process of the cancer cells to migrate from primary tumors to other distant organs, is the cause for ~90% of human cancer death (Chaffer and Weinberg, 2011). Piskounova et al. showed that metastasizing melanoma cells experience severe oxidative stress in the blood and visceral organs, resulting in poor metastases, while supplementations with antioxidants increase tumor metastasis (Piskounova et al., 2015). Le Gal et al. (2015) also showed that the dietary administration of antioxidant NAC increases lymph node metastases in an endogenous mouse model of malignant melanoma. Some human studies also support that intake of antioxidants may cause detrimental effects on cancer development in human subjects (Albanes et al., 1996). Overall, these results support a potential detrimental effect of the antioxidant supplement on tumorigenesis.

There could be many reasons for the inconsistent results: e.g., different antioxidants could have different biological actions and varied effects on IBD and CRC. In addition, some antioxidants can reduce transition metals to a more active state, which can then decompose hydroperoxides into high-energy free radicals. Since, phenolics can act as both antioxidants and prooxidants, it can be difficult to predict their net effects in biological systems (Decker, 1997). Further studies are urgently needed in this area, since many dietary antioxidants are widely consumed by the general public, a better understanding of their effects could lead to a major impact on public health.

## Conclusion

Research by us and others support that the LDEs are increased in the colon tissues of IBD and CRC and play critical roles in promoting the disease development of these two types of diseases. A better understanding of the mode of actions of these compounds could help us to identify novel therapeutic targets of IBD and CRC, helping us to design mechanism-based strategies to reduce the risks of these diseases. In addition, dietary factors, such as LA, heme iron, and antioxidants, could have important implications in regulating the development of IBD and CRC, at least in part, through modulating colonic levels of LDEs. Since these dietary compounds are commonly consumed by the general public, a better understanding of their effects on IBD and CRC could lead to a significant impact on public health.

## Author Contributions

LL and JZ wrote the review. ED and GZ edited the review. All authors contributed to the article and approved the submitted version.

### Conflict of Interest

The authors declare that the research was conducted in the absence of any commercial or financial relationships that could be construed as a potential conflict of interest.
